# How over-parenting impedes individual career exploration: a goal disengagement perspective

**DOI:** 10.1186/s40359-023-01163-w

**Published:** 2023-04-12

**Authors:** Bin Wen, Meng Zhang, Lei Zhang, Yue Zhou, Li Xu

**Affiliations:** 1 HeBei Vocational University of Technology and Engineering, XingTai, 054000 People’s Republic of China; 2 International Business School Suzhou, Xi’an Jiaotong-Liverpool University, Suzhou, 215123 People’s Republic of China; 3 School of Management, Shandong University of Traditional Chinese Medicine, Jinan, 250355 People’s Republic of China

**Keywords:** Over-parenting, Career exploration goal disengagement, Career exploration, Need for parental approval, Goal disengagement perspective

## Abstract

Individuals’ early experiences can shape their lifelong development. Notably, healthy parenting experiences will build a good foundation for successful development, whereas inappropriate parenting experiences hinder healthy development. From the goal disengagement perspective, we propose that over-parenting can elicit individual goal disengagement in the development process, which hinders goal-pursuit behaviors. Data collected from 536 university students from China at three time points supported our hypotheses. Specifically, over-parenting promotes more career-exploration goal disengagement, inhibiting career-exploration behavior. In addition, the process mentioned above is more salient for individuals with a high need for parental approval. The theoretical and practical implications of this research are also discussed.

## Introduction

It is widely acknowledged that individuals’ early experiences play a crucial role in an individual’s development [1]. Parenting experiences in early life are particularly prominent in laying the foundation for individuals’ lifelong development [2]. Precisely, parenting experiences can shape an individual’s psychological and behavioral patterns, exerting a profound impact [3]. Healthy parenting experiences can shape adaptive psychological and behavioral patterns [3], while unhealthy parenting experiences will result in dysfunctional patterns [4]. For instance, parental guidance in career development can help shape individuals’ career adaptability [3, 4].

Over-parenting (sometimes called helicopter parenting) is a highly publicized and controversial form of parenting [4]. It is characterized as the extreme assistance or engagement behaviors provided by parents with the good intention of enhancing their child’s current or future success [5]. However, for its effectiveness, researchers have drawn some inconsistent conclusions [6–8]. On the one hand, the involvement of over-parenting can provide support for children’s development [6–8]. On the other hand, the involvement can also impair children’s autonomy [6–8]. In addition, college students who reported having over-controlling parents reported significantly higher levels of depression and less satisfaction with life [8]. Therefore, considering the inconsistency of previous studies, we need to further explore over-parenting’s underlying mechanism and boundary conditions.

Previous investigation on over-parenting have mainly focused on children’s mental development, such as perceived competence [9], emotional functioning [10], depression and less satisfaction with life [8]. Little attention has been paid to the behavioral sides [11]. Proactive behavioral patterns are essential to individual development. Regarding individual lifelong development, career development is a vital aspect ([12]. Moreover, good career development highly relies on individual proactive career exploration behaviors [13]. Thus, this study will explore how and when over-parenting shape children’s career exploration behavior.

**Fig. 1:**
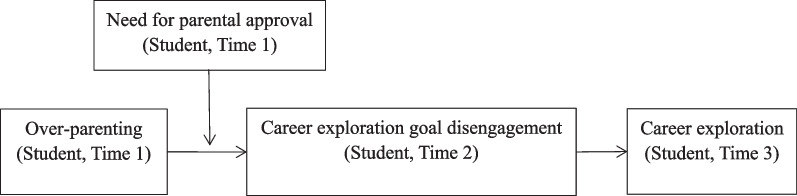
Conceptual framework

Goal disengagement describes people’s attempts to distance themselves from a certain goal [14]. Disengaging from a goal, individuals will dissolve their cognitive, affective, and behavioral commitment [15]. Goal disengagement often occurs after an individual’s goal pursuit is frustrated [15]. Based on a goal disengagement perspective [14, 16], we propose that over-parenting contains too much involvement in individuals’ autonomous development [17], which blocks individual autonomous goal pursuit. Individuals will gradually become over-reliant on their parents psychologically and behaviorally [18]. They are more likely to avoid their responsibilities and transfer them to their parents. Specifically, individuals bearing over-parenting are more likely to pertain higher career exploration goal disengagement [8] and engage in less career exploration behavior [19]. In addition, when individuals are sensitive to the support from their parents [20], the impact of parenting style will be greater. Thus, we further propose that the above-mentioned process will be stronger when individuals pertain a high need for parental approval. In sum, we propose a moderated mediation model to reveal the underlying mechanism and boundary conditions of over-parenting (shown in Fig. 1).

This research will make several contributions. First, we reveal over-parenting’s underlying mechanism of career exploration goal disengagement [15, 16]. Enriching the underlying mechanism deepens the understanding of over-parenting. Second, we incorporate the need for parental approval [20] as the boundary condition. Individuals might pertain different sensitivity towards their parenting experiences and thus react differently. Third, we extend a goal disengagement perspective [15, 16] in understanding over-parenting. Previous research on over-parenting mainly focuses on the self-determination perspective [21] to understand how over-parenting blocks children’s psychological needs. We take a further step and propose that children will not only be frustrated in their goal pursuit but also disengage from it. Fourth, this research help to enrich the outcomes of over-parenting [22]. Previous studies of over-parenting mainly focus on the mental health impact on children [23, 24]. We extend over-parenting’s impact on individuals’ career development, a vital aspect of individuals’ lifelong development. Finally, we also enrich more antecedents of career exploration behaviors [19, 25]. Previous research on career exploration mainly focuses on individual internal attributes, such as personality. We extend the influence of individuals’ parenting experiences on career exploration [26].

## Theoretical background and hypotheses

### Over-parenting, career exploration goal disengagement, and career exploration

Over-parenting has recently drawn more attention from scholars and the general public [11]. Over-parenting is commonly used to describe those parents’ excessive involvement or help actions that are inappropriate for their children’s development despite their original intentions to improve their children’s present or future success [5, 10]. From the definition, we can tell that there are two sides of over-parenting. On the one hand, the original intentions of over-parenting are to help their children to succeed [9]. On the other hand, the involvement or help of over-parenting is excessive and inappropriate for the children [6, 27]. Hence, it is natural for previous studies to draw inconsistent conclusions.

To understand the effectiveness of over-parenting, some researchers draw from self-determination theory [21] and explain how over-parenting’s excessive involvement hinders children’s psychological needs. Specifically, self-determination theory [21] highlights individual intrinsic motivation. For instance, some researchers propose that over-parenting will block children’s autonomy development [7]. A parent’s over-controlling behaviors could signal to the children that the external environment the children live in is threatening or uncontrollable. Then, when over-parenting parents intervene in children’s environment, with the intention to control everything to secure children’s present or future success, children may learn that they are unable to deal with that situation by themselves. Gradually, children’s confidence and perceived competence in themselves will diminish [17].

Going further, we base on a goal disengagement perspective [15, 28] and propose that over-parenting will not only impair children’s autonomy and competence but also elicit children’s goal disengagement. Specifically, over-parenting can cause individuals to develop a dependence on their parents [22]. When faced with difficulties and challenges, individuals will unconsciously develop an avoidance mentality, expecting their parents to step in to solve their problems. Moreover, such effects will be more salient in the effort-demanding goal-pursuit process. Individuals need to put in many efforts and overcome many hardships in the process of goal pursuit, which make goal pursuit a challenging process [15]. However, when over-parenting parents step into individuals’ goal-pursuit process and take on too much responsibility for their children, children will be more likely to disengage from their goal pursuit and put in less effort [14, 15].

Career development is essential in individual development [12, 29]. Career exploration is an essential foundation for individuals’ successful career development. Career exploration involves purposive behavior and cognition that afford access to information about individuals themselves and various occupations [30]. Within the exploration process, individuals can gather career information from two primary sources: the environment and oneself [31]. The external exploration of the environment involves the proactive search for opportunities. The inner exploration of the self involves deeper self-assessment, which can help individuals know themselves more clearly. In this vein, career exploration is also an effort-demanding process [32]. Taken together, we propose that the excessive involvement of over-parenting may result in individuals’ disengagement in effortful career exploration and engage in less career exploration behavior:

H1: Career exploration goal disengagement mediates the negative relationship between over-parenting and career exploration.

### Moderating role of need for parental approval

The need for social approval refers to individuals’ desire to be recognized and favored by relevant others [20]. Since the parents are the most important others in an individual’s development [33], we focus on individuals’ need for parental approval. Individuals with a high need for parental approval lack self-confidence and depend more upon their parents’ opinions. Moreover, individuals with a high need for parental approval will act in ways that they think will ensure the approval of their parents [34]. In contrast, individuals with a low need for parental approval are less concerned with gaining approval from their parents. Moreover, their attitudes and behaviors are less influenced by their parents’ opinions.

Hence, faced with over-parenting [5], individuals with a different need for parental approval will react differently. For individuals with a need for parental approval, the influence of over-parenting will be stronger. When faced with over-parenting’s excessive involvement, individuals with a need for parental approval are more likely to accept parental interventions to secure approval from their parents. Since they place a high value on attaining parental approval, they depend more on their parents. When faced with effort-demanding goal pursuit, they are more likely to disengage [15] and rely on their parents. In contrast, individuals with a low need for parental approval place a low value on attaining parental approval. They can act more independently and make their own decisions. When faced with effort-demanding goal pursuit, they are more likely to keep fully engaged. Thus, we propose:

H2: Need for parental approval moderates the relationship between over-parenting and career exploration goal disengagement, such that the positive relationship will be stronger for students with higher need for parental approval.

### An integrated model

Taken together, we take a goal disengagement perspective [15] to understand the influence of over-parenting. Specifically, we propose that the excessive control and involvement of over-parenting in children’s autonomous development [6, 27] will elicit children’s goal disengagement and be less engaged in goal pursuit. Specifically, over-parenting will inhibit children’s career exploration [32] via career exploration goal disengagement [28]. In addition, children might place different values on the recognition and approval of their parents and react differently towards over-parenting. Under over-parenting, individuals with a high need for parental approval [20] are more likely to disengage in career exploration and conduct less career exploration behaviors:

H3: Need for parental approval moderates the indirect negative relationship between over-parenting and career exploration, such that the relationship will be stronger for students with higher need for parental approval.

## Methods

### Participants and procedure

We collected survey data from a Chinese university. We issued 800 surveys and finally obtained 536 valid university students’ responses. We collected data at three time points with a two-week interval to reduce the common method bias. We used student ID as the match code for students’ multiple responses. At Time 1, university students reported their basic information (i.e., age, gender, their major), experienced over-parenting, and need for parental approval. At Time 2, students reported their career exploration goal disengagement. Finally, at Time 3, they reported their career exploration behavior. Among our final valid sample, 273 (50.90%) students were male, with an average age of 20.29 (*SD* = 1.94). Furthermore, 434 (81.00%) students majored in natural science.

### Measures

All of the responses were rated on a five-point Likert scale, ranging from 1 (*not at all*) to 5 (*completely*). We followed Brislin’s [35] translation-back-translation procedure to translate English scales into Chinese versions.

#### Over-parenting

We measured over-parenting with seven items used by Liu et al. from LeMoyne and Buchanan [5, 11]. Sample item includes “My parents often stepped in to solve life problems for me” (α = 0.86).

#### Need for parental approval

We measured need for parental approval with five items adopted from Crocker et al. [20]. Sample item includes “My self-esteem depends on the opinions my parents hold of me” (α = 0.83).

#### Career exploration goal disengagement

We measured career exploration goal disengagement with four items based on Wrosch and colleagues’ goal disengagement measurement [16]. Sample item includes “For career exploration, it’s easy for me to reduce my effort toward the goal” (α = 0.90).

#### Career exploration

We measured career exploration with eleven items from Stumpf and colleagues [31]. Participants reported their recent career exploration behavior, such as “investigated career possibilities (environment exploration)” and “reflected on how my past integrates with my future career (self-exploration)” (α = 0.92).

## Results

### Preliminary analysis

The hypothesized four-factor model fit well (*χ* ^2^(183) = 852.67, *χ* ^2^/*df* = 4.66, CFI = 0.94, TLI = 0.93, SRMR = 0.07, RMSEA = 0.06) and was significantly better than other alternative models (see Table 1), showing good discrimination validity. These results showed that there are no severe common method biases.

**Table 1:** Confirmatory factor analysis (*N* = 536)

Model	*χ* ^2^	*df*	*χ* ^2^ /*df*	CFI	TLI	SRMR	RMSEA
Four-factor model	852.67	183	4.66	0.94	0.93	0.07	0.06
Three-factor model ^a^	1514.68	186	8.14	0.79	0.76	0.12	0.12
Three-factor model ^*b*^	2079.36	186	11.18	0.70	0.66	0.12	0.14
Three-factor model ^c^	1637.46	186	8.80	0.77	0.74	0.11	0.12
One-factor model ^d^	4132.04	189	21.86	0.38	0.31	0.18	0.20

Four-factor model consists of over-parenting, need for parental approval, career exploration goal disengagement, and career exploration

^a^Combine over-parenting and need for parental approval into one latent factor

^b^Combine career exploration goal disengagement and career exploration into one latent factor

^c^Combine over-parenting and career exploration goal disengagement into one latent factor

^d^Combine all factors into one latent factor

Table 2 presents the descriptive analysis results of all variables. As shown in Table 3, over-parenting was positively correlated with career exploration goal disengagement (*r* = 0.39, *p* < 0.01). Career exploration goal disengagement was negatively correlated with green purchase behavior (*r* = -0.36, *p* < 0.01). The correlation results provided initial support for our hypotheses.

**Table 2:** Means, SD, correlations among variables (*N* = 536)

	*M*	*SD*	1	2	3	4	5	6	7
1 Gender	0.49	0.50	–						
2 Age	20.29	1.94	− 0.09^*^	–					
3 Major	0.19	0.39	0.04	− 0.00	–				
4 Over-parenting	2.70	0.71	− 0.07	− 0.07	0.03	(0.86)			
5 Need for parental approval	3.37	0.68	0.01	− 0.06	− 0.04	0.30^**^	(0.83)		
6 Career exploration goal disengagement	2.83	0.64	0.01	− 0.02	− 0.00	0.39^**^	0.18^**^	(0.90)	
7 Career exploration	3.62	0.58	0.01	− 0.01	− 0.11^**^	− 0.19^**^	0.10^*^	− 0.36^**^	(0.92)

*M* = Mean, *SD* = Standard deviation. Gender: 0 = male, 1 = female. Major: 0 = natural science, 1 = social science. *p < 0.05, **p < 0.01, ***p < 0.001, Cronbach’s alpha in parentheses

**Table 3:** Regression results (*N* = 536)

	Model 1	Model 2	Model 3
	Estimate(*SE*)	Estimate(*SE*)	Estimate(*SE*)
DV: Career exploration goal disengagement			
*Intercepts*	2.98***(0.30)	1.62***(0.32)	1.71***(0.31)
Gender	0.02(0.06)	0.05(0.05)	0.05(0.05)
Age	− 0.01(0.01)	0.00(0.01)	0.00(0.01)
Major	− 0.01(0.07)	− 0.02(0.07)	− 0.01(0.06)
Over-parenting		0.34***(0.04)	0.29***(0.04)
Need for parental approval		0.06(0.04)	0.07(0.04)
Over-parenting × Need for parental approval			0.17***(0.04)
R^2^	0.00(0.00)	0.16***(0.03)	0.19***(0.03)

***p < 0.001

### Hypotheses test

We applied the SPSS macro application from Hayes [36] with a bootstrap approach to obtain confidence intervals for the hypotheses testing. The mediation test indicated that over-parenting significantly correlated with career exploration through career exploration goal disengagement (Estimate = -0.11, *SE* = 0.02, 95%CI = [-0.15, -0.07]), supporting H1.

**Fig. 2:**
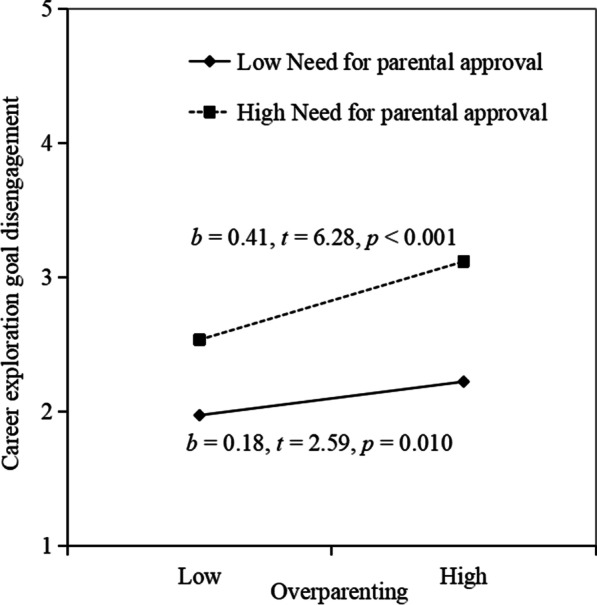
The moderating effects of Need for parental approval

Then, we conducted hierarchical regression to verify the moderating role of need for parental approval. As shown in Table 3, need for parental approval significantly moderated the relationship between over-parenting and career exploration goal disengagement (*b* = 0.17, *SE* = 0.04, *p* < 0.001), supporting H2. As presented in Fig. 2, the relationship between over-parenting and career exploration goal disengagement is stronger for students with higher need for parental approval (*b* = 0.41, *t* = 6.28, *p* < 0.001; 1 SD above the mean) than for students with low need for parental approval (*b* = 0.18, *t* = 2.59, *p* = 0.010; 1 SD below the mean).

Further, the overall moderated mediation test indicated that need for parental approval significantly moderated the indirect relationship between over-parenting and career exploration through career exploration goal disengagement, supporting H3. Specifically, the indirect negative influence is stronger for students with higher need for parental approval (effect = − 0.31, *SE* = 0.06, 95%CI = [− 0.44, − 0.19], 1 *SD* above the mean) than lower need for parental approval (effect = − 0.24, *SE* = 0.04, 95%CI = [− 0.33, − 0.15], 1 *SD* below the mean; Estimated difference = − 0.07, *SE* = 0.02, 95%CI = [− 0.11, − 0.03]).

## Discussion

Through a time-lagged survey study with university students, we found that over-parenting was negatively related to career exploration behaviors through students’ career exploration goal disengagement. Notably, such negative relationship was stronger when students pertained high need for parental approval. This research makes several theoretical and practical implications.

### Theoretical implications

This study makes several theoretical contributions. First, linking over-parenting to career exploration, we reveal the underlying mechanism of career exploration goal disengagement [15, 28]. Specifically, over-parenting will promote individuals to disengage in the goal-pursuit process and put less effort into pursuing the goal, especially for those effort-demanding goals. Since career exploration involves individuals’ purposeful information seeking and requires long-term persistence [31], over-parenting parents might intervene in individuals’ affairs and make decisions for them. Thus, individuals are likely to disengage and transmit the obligations to their over-parenting parents. Enriching the underlying mechanism also contributes to a deeper understanding of over-parenting.

In addition to revealing the underlying mechanism, we also incorporated the boundary condition of need for parental approval [20]. Individuals with different values placed on their parents’ treatment will react differently towards over-parenting. Extending the boundary conditions also contributes to a deeper understanding of over-parenting.

Third, we enrich a goal disengagement perspective [15] in over-parenting research. Previous research on over-parenting mainly takes a self-determination perspective [21]. Based on this, we take a further step to draw on a goal disengagement perspective to explore the effectiveness of over-parenting. Specifically, facing over-parenting’s excessive involvement, children will disengage from their goals and put less effort into goal pursuit.

Fourth, this research expands the impact of over-parenting [22] on children’s career development [19]. Over-parented children will rely more on their parents and avoid their own responsibilities [27]. In this research, we found that over-parenting will encourage children to disengage from their goal pursuit [15], specifically, career exploration goal pursuit. After parents’ excessive intervention, children will become more passive in their career development, avoid things they need to do themselves, and shift their obligations to parents. In this aspect, we enrich the detrimental effects of over-parenting on individuals’ career development [19].

Finally, we contribute to more antecedents of career exploration behaviors [37]. Career exploration plays a crucial role in individuals’ lifelong career development. Early career exploration provides a good foundation for smooth development later in life [38]. Researchers have endeavored a lot to reveal how to promote career exploration behaviors. We take a more systematic perspective, incorporate individuals’ parenting experiences into research, and reveal how over-parenting inhibits career exploration behaviors.

### Practical implications

First, this research highlights the negative influence of over-parenting. Over-parenting can negatively influence children’s autonomy and competence, which harms children’s healthy development [8]. This study warns parents to reflect on the appropriateness of their parenting style [39]. While parents often have good intentions, excessive involvement and intervention are not conducive to children’s healthy long-term development. With overprotective parents, children will lack opportunities to experiment, gain experience from failures or setbacks, and learn from them [22]. Parents need to be aware of the detrimental effects of over-parenting. Parents can communicate frequently with their children to seek children’s perspectives.

Second, this research highlights the detrimental effects of goal disengagement [15]. Goal pursuit is often an effort-consuming process. Lack of sufficient commitment often leads to failure in goal pursuit. In pursuing personal development goals, individuals should remain proactive, improve persistence, and avoid disengagement [28]. Academic advisors and career services on campus can provide training to foster college students’ intrinsic motivation in order to achieve their career goal, such as providing intervention programs to cultivate college students in navigating parents’ intrusiveness and in promoting college students’ agency.

Third, this research highlights the importance of career exploration [31]. Career exploration builds the foundation for individuals’ lifelong development. Individuals ought to place a high value on career exploration [19]. Career exploration requires individuals to actively search and gather information about internal and external sources [37]. It is a time-consuming and energy-intensive process that requires sustained, long-term persistence. Teachers can provide more career exploration guidance for students to provide more favorable environments.

Finally, children should view parental approval cautiously [20]. Parental approval can sometimes be biased and misguiding. Individuals ought to take a holistic view on parents’ involvement and seeking parental approval in individual development. Forming one’s independence is an important developmental task for individuals. Individuals should avoid excessive dependence on parents and be cautious about parental involvement and participation [22]. Additionally, educators and policy makers may develop intervention programs for parents and teachers to inhibit intensive care-giving and to promote autonomy supportive parenting.

### Limitations and future directions

First, only one survey study is insufficient for drawing strong casual conclusions. Future research can supplement more research designs to ensure internal and external validity, such as longitudinal and cross-lagged designs [4, 40]. To supplement qualitative in-depth investigations can also contribute to a deeper understanding. In addition, we only conducted our study with Chinese participants. For over-parenting, there might be some cultural differences [41]. Future research could supplement more cross-cultural comparison designs. Regarding cultural factors, filial piety [42] may be a unique culturally specific belief, guiding individual need for parental approval. Meanwhile, future research could expand more theoretical perspectives to explore the underlying mechanisms of over-parenting [22]. In addition, future research may incorporate more control variables to rule out alternative explanations, such as the parents’ education, employment, income, and family structures. Finally, as for over-parenting’s impact in career aspect, future research could incorporate other aspects of career development [43]. For instance, future research could combine a longitudinal design [4] to incorporate career success [43] into research.

## Conclusion

Parenting experiences can exert a profound influence on individuals’ lifelong development. Through a survey study with Chinese university students, we find that over-parenting can promote more career exploration goal disengagement and result in less career exploration behavior. In particular, it is more salient for individuals with a high need for parental approval. We hope our research can inspire more research on over-parenting and its outcomes.

## Data Availability

Data are available from the corresponding author upon reasonable request.
